# Supplementary material for: A Longitudinal and Comparative Content Analysis of Instagram Fitness Posts
Source: Int J Environ Res Public Health. 2022 Jun 3;19(11):6845. doi: 10.3390/ijerph19116845 (PMC9180174; doi:10.3390/ijerph19116845)
Supplement: Supplementary file 1 [file ijerph-19-06845-s001.zip › ijerph-1683217-supplementary.pdf]

A longitudinal and comparative content analysis of Instagram fitness posts

Supplementary Material

Table S1. Instagram fitness accounts from 2019 and 2021 used in the analysis.

2019		2021	
Influencers	Brands	Influencers	Brands
Anllela Sagra	Adidas	Sommer Ray	Nike
Jen Selter	Lululemon	Michelle Lewin	Adidas
Julia Gilas	Vans	Kayla Itsines	Puma
Kayla Itsines	Nike	Anllela Sagra	Under Armour
Lauren Drain	Pure Gym	Ana Cheri	New Balance
Maria Sharapova	My Protein	Jen Selter	Gymshark
Michelle Lewin	Anytime Fitness	Issa Vegas	The North Face
Paige Hathaway	Gymshark	Eva Andressa	Patagonia
Sandra Prikker	Under Armour	Sascha Barboza	Reebok
Serena Williams	Red Bull	Paige Hathaway	Lululemon

Table S2. Final Coding Guide with Explanatory Comments.

**Variable**	**Label**	**Comments**
**User Name**		- Instagram handle
**Image Type**		
Visual	1. Still image<br>2. Video content<br>3. Photo Series	
**Image Content**		
Category	1. People<br>2. Other	
Gender	1. Female<br>2. Male<br>3. N/A	
Ethnicity	1. White<br>2. Mixed<br>3. Asian<br>4. Black, African, Caribbean<br>5. Other Ethnic Group<br>7. Unclear<br>8. N/A	- Will be used descriptively only. To be researched where possible rather than deduced from image<br>- Code N/A only if the image is not of a person
Age	1. 16-20<br>2. 21-25<br>3. 26-30<br>4. 31-35<br>5. 36-40<br>7. Unclear<br>8. N/A	- Will be used descriptively only. To be researched where possible rather than deduced from image

Variable	Label	Comments
Location	1. Fitness space<br>2. Residential property<br>3. Indoor other<br>4. Outdoor other<br>5. Unclear	- Any space that can be used as a fitness space (e.g., gym or swimming pool) to be coded as a fitness space regardless of whether it is being used in this way
Equipment	1. Exercise equipment present<br>2. No exercise equipment	
**Image Purpose**		
Promotional	1. Yes: fitness-related<br>2. Yes: appearance-related<br>3. Yes: other<br>4. No	- Avoid making assumptions based on what is known about the influencer
Educational	1. Yes: fitness-related<br>2. Yes: appearance-related<br>3. Yes: other<br>4. No	- Videos showing exercises: no explanatory caption is needed for these to be considered educational<br>- In the case of images, an explanatory caption/text of some kind is needed for them to be categorised as educational
Motivational	1. Yes: fitness-related<br>2. Yes: appearance-related<br>3. Yes: other<br>4. No	- Emphasis on transformation is generally considered motivational<br>- Take caption into account
**Body Depiction**		
Thinness	1. Low body fat<br>2. Not low body fat<br>3. Body not shown<br>4. N/A	
Muscularity	1. Little to no definition<br>2. Visible definition<br>3. High-level definition<br>4. Body not shown<br>5. N/A	- High level vs. visible definition: Avoid being influenced by other pictures. Take staging into account (e.g., lighting, pose)
Face visibility	1. Face visible<br>2. Face not visible<br>3. N/A	- Over 50% of face must be visible to meet criteria for 'face visible'
Glutes in focus	1. Yes<br>2. No<br>3. N/A	- Code what is emphasised in the image, not simply what is visible<br>- If a body part is not in the picture, code no and not N/A
Abs in focus	1. Yes<br>2. No<br>3. N/A	

Variable	Label	Comments
Arms in focus	1. Yes<br>2. No<br>3. N/A	
Chest in focus	1. Yes<br>2. No<br>3. N/A	
Legs in focus	1. Yes<br>2. No<br>3. N/A	
Proportion of body visible	1. 100%<br>2. 75%<br>3. 50%<br>4. 25%<br>5. N/A	- 100% only if full body (head to toes)<br>- Code as 0.25, 0.5, 0.75, 1 instead of with %
**Pose**		
Active	1. Yes<br>2. No<br>3. N/A	
Glutes pushed towards camera	1. Yes<br>2. No<br>3. N/A	- Pose needs to show some intentionality (i.e., cannot be primarily due to clothing/body shape)
Back arched	1. Yes<br>2. No<br>3. N/A	
Wide stance	1. Yes<br>2. No<br>3. N/A	
Pulling at clothing/hair	1. Yes<br>2. No<br>3. N/A	- Touching hair counts as pulling
One foot forward to emphasise glutes	1. Yes<br>2. No<br>3. N/A	
Pouting	1. Yes<br>2. No<br>3. N/A	- Can be both open-mouthed and pouting
Direct gaze	1. Yes<br>2. No<br>3. N/A	
Mouth open	1. Yes<br>2. No<br>3. N/A	- Does not include smiling<br>- Not mutually exclusive with other mouth variables.<br>- If tongue is visible, mouth is always considered open

Variable	Label	Comments
Biting lip/tongue	1. Yes<br>2. No<br>3. N/A	
**Clothing**		
Type of clothing	1. Activewear<br>2. Sexualised<br>3. No visible clothing<br>4. Everyday clothing<br>5. N/A	- Activewear: must look like one could exercise in it
Fit of clothing	1. Tight fitting<br>2. Standard fit<br>3. Comfortable fit<br>4. Loose fit<br>5. Swimsuit/underwear<br>6. N/A	- Code swimsuit/underwear if one piece of clothing (top or bottom) fits in these categories
Skin exposure	1. Not revealing<br>2. Moderately revealing<br>3. Very revealing<br>4. Extremely revealing<br>5. N/A	

Table S3. Inter-Rater Reliability for Coding Variables.

Variable	Reliability (Kappa)
Image type	
Visual	.93
Image content	
Category	.94
Gender	.89
Ethnicity	–
Age	–
Location	.76
Equipment	.82
Image purpose	
Promotion	.69
Education	.71
Motivation	.47
Body depiction	
Thinness	.81
Muscularity	.66
Face visibility	.87
Glutes in focus	.87
Abs in focus	.85
Arms in focus	.77
Chest in focus	.87
Legs in focus	.75
Proportion of body visible	.81
Pose	
Active	.88
Glutes pushed towards camera	.82
Back arched	.84

Variable		Reliability (Kappa)
	Wide stance	.72
	Pulling at clothing/hair	.89
	One foot forward to emphasise glutes	.75
	Pouting	.72
	Direct gaze	.86
	Mouth open	.76
	Biting lip/tongue	.95
Clothing		
	Type of clothing	.84
	Fit of clothing	.74
	Skin exposure	.63

Table S4. Observed Frequencies for Demographic Characteristics and Image Content Variables.

	Variable (sample size)	Whole data set<br>*n* (%)	Influencers 2019<br>*n* (%)	Brands 2019<br>*n* (%)	Influencers 2021<br>*n* (%)	Brands 2021<br>*n* (%)
**Visual (*N*=400)**	Still Image	162 (40.5)	64 (39.5)	48 (29.6)	27 (16.7)	23 (14.2)
	Video content	163 (40.8)	23 (14.1)	48 (29.4)	49 (30)	43 (26.4)
	Photo series	75 (18.8)	13 (17.3)	4 (5.3)	24 (32)	34 (45.3)
**Category (*N*=400)**	People	348 (87)	86 (24.7)	86 (24.7)	96 (27.6)	80 (23)
	Other	52 (13)	14 (27)	14 (27)	4 (7.7)	20 (38.3)
**Gender (*n*=348)**	Female	269 (77.3)	84 (31.3)	40 (14.9)	95 (35.3)	50 (18.5)
	Male	72 (20.7)	2 (2.8)	43 (59.7)	1 (1.4)	26 (36.2)
	Unable to code	7 (2)	0 (0)	3 (42.9)	0 (0)	4 (47.1)
**Ethnicity (*n*=348)**	Asian	11 (3.2)	0 (0)	2 (29)	0 (0)	9 (81)
	Black/African American	55 (15.8)	8 (14.5)	21 (38.1)	0 (0)	26 (47.3)
	Hispanic/Latinx	70 (20.1)	20 (28.6)	0 (0)	50 (71.4)	0 (0)
	White	164 (47.1)	58 (35.4)	43 (26.2)	38 (23.2)	25 (15.2)
	Other	35 (10.1)	0 (0)	12 (34.3)	8 (23)	15 (43)
	Unable to code	13 (3.7)	0 (0)	8 (61.5)	0 (0)	5 (38.5)
**Age (*n*=348)**	16-20	6 (1.7)	0 (0)	4 (66.7)	0 (0)	2 (33.3)
	21-25	89 (25.6)	15 (16.9)	30 (33.7)	21 (23.6)	23 (25.8)
	26-30	108 (31)	22 (20.4)	25 (23.1)	27 (25)	34 (31.5)
	31-35	93 (26.7)	41 (44)	17 (18.3)	28 (30.1)	7 (7.5)
	36-40	36 (10.3)	8 (22.2)	1 (2.8)	19 (52.8)	8 (22.2)
	Unable to code	16 (4.6)	0 (0)	9 (56.3)	1 (6.3)	6 (37.5)
**Location (*N*=400)**	Fitness space	101 (25.3)	19 (18.8)	40 (39.6)	24 (23.8)	18 (17.8)
	Residential property	44 (11)	20 (45.5)	0 (0)	21 (47.7)	3 (6.8)
	Indoor other	72 (18)	13 (18)	15 (20.8)	20 (27.7)	24 (33.3)
	Outdoor other	143 (35.8)	38 (26.6)	35 (24.5)	34 (23.8)	36 (25.2)
	Unable to code	40 (10)	10 (25)	10 (25)	1 (2.5)	19 (47.5)

Table S5. *Distribution of the Fit Ideal by Account Type and Year.*

**Account Type**	**Year**			
	**2019**		**2021**	
	n	**%**	***n***	**%**
**Influencer**				
Yes – Fit Ideal	58	67.4	65	67.7
No – Fit Ideal	28	32.6	31	32.3
Total – Fit Ideal	86	100	96	100
**Brand**				
Yes – Fit Ideal	29	33.7	17	21.3
No – Fit Ideal	57	66.3	63	78.8
Total – Fit Ideal	86	100	80	100

Table S6. *Distribution of Sexualisation by Account Type and Year.*

Account Type	Year			
	2019		2021	
	n	%	*n*	%
**Influencer**				
Yes – Sexualisation	28	32.6	36	37.5
No – Sexualisation	58	67.4	60	62.5
Total – Sexualisation	86	100	96	100
**Brand**				
Yes – Sexualisation	1	1.2	0	0
No – Sexualisation	85	98.8	80	100
Total – Sexualisation	86	100	80	100

Table S7. *Distribution of Objectification by Account Type and Year.*

Account Type	Year			
	2019		2021	
	n	**%**	***n***	**%**
**Influencer**				
Yes - Objectification	12	14	27	28.1
No - Objectification	74	86	69	71.9
Total - Objectification	86	100	96	100
**Brand**				
Yes - Objectification	9	10.5	11	13.8
No - Objectification	77	89.5	69	86.2
Total - Objectification	86	100	80	100
